# Supplementary material for: WordCommentsAnalyzer: A windows software tool for qualitative research
Source: F1000Res. 2018 Sep 4;7:536. Originally published 2018 May 3. [Version 2] doi: 10.12688/f1000research.14819.2 (PMC6137411; doi:10.12688/f1000research.14819.2)
Supplement: Supplementary file 3 [file f1000research-7-17614-s0002.tgz › b2a79496-4ada-47c4-9682-a99ce867836d.zip!/Eight Iranian Health Journals/Journal of Research in Rehabilitation Sciences _45.docx]

© Journal of Research in Rehabilitation Sciences . This work is licensed under CC BY-NC 4.0 (https://creativecommons.org/licenses/by-nc/4.0/).

Volume & Issue: دوره 7، شماره 1: 1390

Title: تأثير 8 هفته تمرين درماني منتخب و برنامه خود درماني با بروشور روي ميزان قدرت و دامنه حرکتي گردن در مبتلایان به گردن درد مزمن

http://jrrs.mui.ac.ir/index.php/jrrs/article/view/148

چکيده زمينه و هدف: گردن درد يکي از اختلالات شايع در کاربران رايانه مي­باشد.هدف از اين مطالعه بررسي تأثير 8 هفته تمرين درماني منتخب و  برنامه خود درماني با بروشور بر ميزان قدرت و دامنه حرکتي گردن در بيماران با گردن درد مزمن مي­باشد. مواد و روش­ها: جامعه آماري اين تحقيق شامل 66 بيمار مبتلا به گردن درد مزمن باميانگين سني (10/3 ± 19/39سال) بودند.براي سنجش ميزان قدرت ايزومتريک عضلات گردن از دينامومتر دستي (power track commander II)، و براي اندازه­گيري دامنه حرکتي از گونيامتر استفاده شد. براي تحليل داده­ها ابتدا مقادير اختلاف پيش آزمون و پس آزمون محاسبه و سپس از طريق آزمون ANOVA مورد ارزيابي قرار گرفتند)05/0(P<. يافته­ها: نتايج نشان داد بين ميانگين قدرت عضلات گردن سه گروه­ تفاوت معني داري وجود دارد )05/0(P<. مقايسه دامنه حرکتي گردن درگروه­هاي مختلف، حاكي از آن است كه دامنه حركتي گردن گروه تمرين درماني منتخب بجز در حرکت چرخش به چپ، در حرکات ديگر با دامنه حركتي گردن دو گروه ديگر  تفاوت معنا­داري دارد.اما گروه خود درماني با بروشور تنها در حرکت چرخش به راست با گروه کنترل تفاوت معنا­دار داشت )05/0(P<. بحث و نتيجه گيري: بطور كلي مي توان نتيجه گيري نمود كه برنامه تمرين درماني منتخب زير نظر مربي، مي تواند نتايج سودمندتري در افزايش قدرت عضلات و دامنه حرکتي گردن در جهات مختلف نسبت به روش خود درماني با بروشور  داشته باشد.

Title: ارزیابی قوس کف پا و میزان آسیب های غیر برخوردی زانو و مچ در کاراته کاهای مرد

http://jrrs.mui.ac.ir/index.php/jrrs/article/view/149

مقدمه: هدف از انجام این تحقیق بررسی تغییرات قوس کف پا و تأثیر آن در میزان آسیب­های غیر برخوردی زانو و مچ پا در کاراته­کاهای مرد می باشد. مواد و روشها: از بین 288 کاراته­کای حرفه­ای با کمربند مشکی و بالاتر در استان کرمان 45 نفر که دارای آسیب در ناحیه مفاصل زانو و مچ پا بودند در تحقیق شرکت داده شدند. اطلاعات مربوط به آسیب با استفاده از فرم ثبت اطلاعات جمع آوری شد و قوس کف پا با استفاده از شاخص CSI[1] اندازه گیری شد. از آزمون t مستقل و خی دو[2] برای تجزیه و تحلیل داده ها استفاده شد (p<0.05). یافته ها: بیشترین میزان آسیب ها در ناحیه زانو (2/62 درصد) و در زمان تمرین (3/82 درصد) گزارش شد و شدت آسیب در اکثر موارد (8/57 درصد) جزئی بوده است. همچنین نتایج نشان داد که قوس کف پا در پای راست در 9/68% افراد آسیب دیده غیرطبیعی و در1/31% افراد آسیب دیده طبیعی و در پای چپ در 8/77% افراد آسیب دیده غیرطبیعی و در 2/22% افراد آسیب دیده طبیعی بود؛ در مجموع بیشترین میزان غیر طبیعی قوس کف پا در پای چپ (8/77 درصد) مشاهده شد. نتایج حاصل از تجزیه و تحلیل آماری نشان داد که بین میزان قوس کف پا در دو پای راست و پای چپ ارتباط معنی داری وجود ندارد. و نیز بین میزان قوس کف پا و شدت آسیب ارتباط معنی داری مشاهده نشد، همچنین تجریه و تحلیل داده ها نشان داد که بین میزان آسیب­های غیربرخوردی اندام تحتانی بر اساس میزان قوس کف پا اختلاف معنی داری وجود دارد. نتیجه گیری: نتایج تحقیق حاضر نشان می دهد که غیر طبیعی بودن میزان قوس کف پا احتمالاً میزان آسیب های غیر برخوردی اندام تحتانی در کاراته کاهای مرد را تحت تأثیر قرار می دهد.   CSI،

Title: تاثير اسپلينت دورسال مچ دست بر سندرم تونل كارپال بر اساس مطالعه هدایت عصب حرکتی مدين

http://jrrs.mui.ac.ir/index.php/jrrs/article/view/150

زمينه و هدف : سندرم تونل كارپال يكي از شايع‌ترين نوروپاتيهاي اندام فوقاني است كه در اثر گير افتادن عصب مدين در مچ ايجاد مي‌شود. در اين تحقيق به بررسي نوع جديدي از اسپيلنت(اسپيلنت دورسال مچ دست) در مقايسه با نوع مرسوم (اسپيلنت پالمار) بر اساس پارامترهاي الكترودياگنوزيس پرداخته شده است. روش بررسي: به منظور انجام يك مطالعه نيمه تجربي از نوع كارآزمايي باليني قبل و بعد، 22 بيمار زن مبتلا به CTS خفيف يا متوسط مورد ارزيابي قرار گرفتند.بيماران مبتلا به CTS درمان نشده كه با انجام تست هاي الكترو دياگنوزتيك، مبتلا به نوع خفيف يا متوسط شناخته مي شدند، به طور تصادفي به دو گروه تقسيم شدند. يك گروه (12 نفر)، اسپيلنت دورسال مچ دست و گروه ديگر(10 نفر) اسپيلنت پالمار مچ دست( هر دو در وضعيت نوترال مچ) دريافت نمودند و يك بار قبل از مداخله درماني و بار ديگر به فاصله زماني 4 هفته پس از مداخله درماني، مورد ارزيابي توسط تست هاي الكترودياگنوزتيك قرار گرفتند. متغيرهاي موردبررسي شامل تأخير هدايت حرکتی ديستال عصب مدين، آمپليتود و سرعت هدايت عصب  حرکتی بود. به منظور آناليز داده‌هاي مطالعه از  آزمون هاي  كولمو گروف اسميرونوف، T مستقل و زوج شده استفاده شد. يافته ها : نتايج به دست آمده نشان مي‌دهد كه پارامترهاي هدايت عصب حرکتی مدين در هر دو گروه پالمار و دورسال پس از مداخله 4 هفته اي تغيير قابل ملاحظه اي در جهت بهبود داشته است.(05/0  p<). همچنين مقايسه پارامترهاي الكترودياگنوزيس حرکتی بين دو گروه پس از 4 هفته اختلاف معناداري را نشان نداد. (05/0<p). نتيجه گيري : مطالعه حاضر نشان داد كه به لحاظ علائم الكترودياگنوزيس حرکتی عصب مدين ، هر دواسپلينت دورسال مچ دست و اسپلينت كوكاپ مرسوم در بهبود سندرم تونل كارپال تاثیر مثبت دارند.

Title: جنبه های آکوستیکی صوت در لارنژیت ناشی از ریفلاکس و پولیپ تار صوتی

http://jrrs.mui.ac.ir/index.php/jrrs/article/view/152

مقدمه : آسیب های خوش خیم حنجره ، مشکلات نسبتاً شایعی هستند که اغلب در پی بروز تغییر در صدا مورد بررسی قرار می گیرند. در بررسی صوت علاوه بر ارزیابی ادراکی ، از شیوه های آزمایشگاهی آکوستیک استفاده می شود که به دلیل غیر تهاجمی بودن ، آسان بودن ثبت داده ها و عینی بودن نتایج روش مناسبی می باشد. در انواع اختلالات صوت ، بررسی و مطالعه ویژگی های آکوستیکی ، برای تشخیص و درمان کمک کننده هستند . لذا با توجه به شباهت نسبی ویژگی های شنیداری صوت ناشی از پولیپ و لارنژیت ناشی از ریفلاکس ، این مطالعه برای کشف تفاوتهای آکوستیکی بین آن ها انجام شده است. مواد و روش ها : در این مطالعه توصیفی – تحلیلی ،غیر مداخله ای و گذشته نگر بیماران مبتلا به پولیپ و لارنژیت ناشی از ریفلاکس در دامنه سنی 74-20 سال مورد بررسی قرار گرفته اند . برای بررسی ویژگی های صوت از واکه /æ/ استفاده شد و تجزیه و تحلیل آن ها با استفاده از نرم افزار praat صورت گرفت. یافته ها : میانگین بسامد پایه ، دامنه تغییرات بسامد پایه و آشفتگی شدت در مردان مبتلا به پولیپ بالاتر از لارنژیت ناشی از ریفلاکس بود و دامنه تغییرات بسامد پایه وآشفتگی شدت و نسبت هارمونیک به نویز در دو گروه بیماران تفاوت معنادار داشت ، اما مقایسه میانگین بسامد پایه و آشفتگی بسامد پایه در مردان  مبتلا به پولیپ و لارنژیت ناشی از ریفلاکس وهمه ی ویژگی های صوتی در زنان اختلاف معناداری نشان نداد.  بحث: آشفتگی شدت ، دامنه تغییرات بسامد پایه و نسبت هارمونیک به نویز در مردان مبتلا به پولیپ و لارنژیت ناشی از ریفلاکس متفاوت است و این ویژگی ها در پولیپ بیشتر آسیب می بیند . بنابراین می توانند به عنوان وجه تفاوت گذاری ( تشخیص افتراقی ) بین این دو گروه از بیماران  مورد استفاده قرار گیرند .

Title: يك مطالعه موردي در زمينه طراحي و ساخت ارتز دايناميك توراكولومبار و تاثير آن بر ميزان كاهش قوس كايفوز در بيماري شوئرمن

http://jrrs.mui.ac.ir/index.php/jrrs/article/view/155

مقدمه : اين مطالعه به منظور ارزيابي تاثير نوع جديدي از ارتز توراكولومبار بر روي بيماران مبتلا به كايفوز شوئرمن در دانشگاه علوم پزشكي ايران و با همكاري يك كلينيك ارتوپدي فنی در اصفهان انجام گرفت. اين ارتز نوين، استفاده از يك سيستم دايناميك را نسبت به نوع متداول استاتيك، جهت ترغيب ستون فقرات تراسيك به انجام عمل Ext. ارائه مي نمايد. مواد و روشها : جهت دستيابي به هدف تحقيق از يك ارتز پلاستيكي ستون فقرات كه داراي مفصل Hinge و بصورت دايناميك ساخته شد، جهت درمان بيماران داراي كايفوز شوئرمن با زاويه قوس 45 و 65 درجه به مدت دو ماه استفاده گرديد. از اطلاعات راديوگرافي به همراه اطلاعات موجود در پرسشنامه، جهت ارزيابي تاثير اين ارتز استفاده شد يافته ها : ميزان متوسط زاويه قوس با استفاده از ارتز دايناميك توراكولومبار، كاهش 5/29 درصد را نشان داد كه بسيار نزديك به نتايج حاصل از درمان  با ارتز ميلواكي با كاهش 5/35 درصد بود . بحث : نتايج اوليه نشان داد كه ارتز در جلوگيري از پيشرفت كايفوز شوئرمن موثر بوده و In orthosis correction توسط وسيله، رضايتبخش است. به علاوه مقبوليت ظاهري ارتز قابل مقايسه با ميلواكي نبوده و همين امر باعث استفاده بهتر از ارتز گرديد. كليد وا‍‍ژه ها : ارتز، دايناميك، توراكولومبار، اصلاح قوس، كايفوز شوئرمن

Title: بررسي تاثير یکدوره برنامه تمريني بر ميزان دامنه حركتي گردن و گردن درد افراد غيرورزشكار

http://jrrs.mui.ac.ir/index.php/jrrs/article/view/156

مقدمه: بعد از کمردرد، گردن درد شايع­ترين درد موجود در افراد (بخصوص در بین سالمندان) است. هدف این پژوهش بررسي تاثير یک دوره برنامه تمريني بر ميزان دامنه حركتي گردن و گردن درد افراد غيرورزشكار بود. مواد و روش­ها: 60 آزمودنی سالمند (24 مرد و 36 زن) با میانگین سن 5/4± 56 سال و با میانگین وزن 4/4 ± 71 کیلوگرم بصورت داوطلبانه جهت اجرای این پژوهش انتخاب شدند. برنامه ورزشي اجرا شده شامل تمرينات كششي (سه ماه) و تقويتي (سه ماه) براي عضلات ناحیه گردني همراه با ابزارهای حمایت­گر خارجی در طول دوره درمان بود. برنامه تمرینی با يك ست[1] و هشت تكرار (در جلسه اول) شروع و در جلسه آخر با سه ست و 16 تكرار پايان يافت. براي اندازه­گيري قوس گردني، عكس­هاي راديوگرافي از نماي جانبي گرفته شده و ميزان زاويه لوردوز گردني با استفاده از روش كاب از مهره 1 تا 7 گردني محاسبه گرديد. برای اندازه­گیری انعطاف­پذیری عضلات گردن از دستگاه انعطاف­سنج، از سیستم بصری اندازه­گیری درد برای اندازه­گیری درد، از فلكسومتر ليتون جهت اندازه­گيري دامنه حركتي گردن و از داينامومتر براي اندازه­گيري قدرت عضلات اكستنسور گردن استفاده گردید. جهت تجزیه و تحلیل آماری از آزمون­های تی مستقل و تی زوجی استفاده شد (P≤ 0.05) یافته­ ها: ميزان دامنه حركتي گردن در چهار جهت و ميانگين قدرت عضلات اكستنسور گردن پس از اجرای پروتكل تمرینی در گروه تجربي افزايش قابل ملاحظه­اي پيدا نموده و قوس گردني و نمرات درد آزمودني­ها كاهش يافت بحث ­و ­نتیجه ­گیری: براساس نتایج این پژوهش نتیجه­گیری می­شود احتمالاً بتواند منجر به کاهش گردن درد شود. از مکانیزم­هایی که به موجب آن، گردن درد افراد با برنامه تمرینی کاهش یافته است می­توان به افزایش دامنه حرکتی در گردن، افزایش قدرت عضلات اکستنسور و کاهش زاویه لوردوز گردنی اشاره کرد واژگان ضروری: گردن درد، برنامه تمرینی، لوردوز گردنی

Title: مطالعه مقدماتی بررسی اثربخشی آموزش گروهی والدین کودکان اوتستیک بر دانش و میزان استرس – اضطراب وافسردگی آنها

http://jrrs.mui.ac.ir/index.php/jrrs/article/view/157

مقدمه:مطالعاتی که اثربخشی آموزش به والدین را در گروه کودکان اتیستیک سنجیده اند محدود می باشد. اين پژوهش با هدف بررسي اثر بخشی آموزش گروهي والدين كودكان اوتيستيك بر دانش والدين در مورد بيماري و راه‌هاي كمك به كودك و ميزان استرس ـ اضطراب ـ افسردگي آنان انجام شده است مواد و روشها: دردی ماه 1386از ميان والدين كودكان اوتيستيك كه جهت درمان و توانبخشي فرزند خود به بيمارستان روزبه مراجعه كردند و علاقمند به شركت در جلسات آموزشي گروهي والدين بودند. 12والد در اين مطالعه شركت كردند. جهت گردآوري داده‌ها از برگۀ تاريخچه‌گيري گفتار ـ زبان، شنوايي، برگه سنجش آگاهي والدين، مقياس افسردگي ـ اضطراب ـ استرس والدین و پرسشنامه ميزان رضايتمندي شركت‌كنندگان استفاده شد نتایج: يافته‌ها نشان داد اين دورۀ آموزشي باعث افزايش سطح آگاهي والدين در مورد بيماري و راه‌هاي كمك به كودك شد (P<0.001) و والدين كيفيت اين دوره آموزشي را در حد مطلوب ارزيابي كردند. كاهشی در ميزان اضطراب، افسردگي و استرس والدين به دست نیامد بحث ونتيجه‌گيري:دوره‌هاي آموزشي گروهي والدين می تواند آگاهي والدين رادرمورد کودک و درمان آن به طور معناداري افزايش دهد.مطالعات تکمیلی با نمونه بیشتر و سنین مختلف پیشنهاد می شود

Title: بررسی مقایسه ای جهت مندی ترسیم اشکال در کودکان پیش دبستانی و دبستانی شهر تهران

http://jrrs.mui.ac.ir/index.php/jrrs/article/view/159

مقدمه:  این پژوهش به منظور مقایسه جهتمندی ترسیم اشکال ثابت در کودکان پیش دبستانی و دبستانی با توجه به نقش دست برتری در آنان انجام پذیرفته است .  مواد و روش ها:  در پژوهش مقطعی حاضر، 160 کودک ( 80 دختر و 80 پسر) با میانگین سنی 96/7 در مقاطع پیش دبستانی و دبستانی به صورت تصادفی خوشه ای انتخاب شدند . ابزارهای مورد استفاده آزمون دست برتری چاپمن (1987) و مقیاس جهت ترسیم اشکال ثابت بود . برای تجزیه و تحلیل داده های آماری پژوهش از آزمون مجذور کا استفاده شد.        یافته ها: نتایج نشان داد که  بین جهتمندی ترسیم اشکال ثابت با دست برتری رابطه وجود دارد (001/0=P)و نیز جهتمندی ترسیم اشکال ثابت در کودکان پیش دبستانی و دبستانی متفاوت است (009/ ، 007/0=P) همچنین نتایج نشان داد که جهتمندی ترسیم اشکال ثابت در کودکان پیش دبستانی و دبستانی تحت تاثیر دست برتری است (009/ ، 010/0=P) .  بحث: راست دستي يا چپ دستي از عوامل تأثيرگذار بر روي عملكردها و عادات و حركات افراد بوده و از آنجاییکه راست دست ها و چپ دست ها بازنمایی متفاوتی از دست خود دارند ، بنابراین با دو ساختار و راهبرد متفاوت شناختی در چپ دست ها و راست دست ها مواجه هستیم . اين تحقيق كاربردهاي مهمي در تشخيص مشكلات رسم اشكال و همينطور نگارش حروف الفباي فارسي دارد، پیشنهاد می شود با تداوم چنين تحقيقاتي در گروههاي خاص از جمله نابينايان،افراد كم توان ذهني وافراد با ناتواني هاي يادگيري بتوان به الگوهاي بهنجار ونابهنجار ترسيم اشكال در كودكان ايراني دست يافت.

Title: بررسی عوارض بلند مدت ناشی از قطع عضو و میزان رضایت از پروتز در جانبازان، با قطع عضو بالای زانو یک طرفه در استان تهران

http://jrrs.mui.ac.ir/index.php/jrrs/article/view/160

مقدمه: آمپوتاسيون يکي از مختل کننده ترين و آسيب رسان ترين وقايع احساسي است که يک فرد ممکن است در زندگي خود تجربه کند. افرادی با قطع عضو پس از دریافت پروتز، الگوهایی از راه رفتن را اتخاذ می کنند که سبب ایجاد دردهایی  مثل درد کمر، درد مفصل ران، درد در پای مقابل می شود. با وجود شیوع بالای دردها در افرادی با قطع عضو، آمار دقیقی از آن ها در جانبازان جنگ تحمیلی در دست نیست. در حالیکه برای بهبود درد ممتد در افرادی با قطع عضو نیاز به برنامه های توانبخشی و درمانی موثر است. هدف: بررسی عوارض بلند مدت ناشی از قطع عضو و میزان رضایت  از پروتز در جانبازان با قطع عضو بالای زانوی یک طرفه در استان تهران در سال 1387 مواد و روش ها: این مطالعه از نوع توصیفی-مقطعی بوده و بر روی 61 نفر از جانبازان با قطع یک طرفه اندام تحتانی از بالای زانو، روی زانو و لگن استان تهران انجام گرفت. روش جمع آوری داده ها با استفاده از ترجمه فارسی پرسشنامه Prosthesis Evaluation Questionnaire بوده که روایی و پایایی آن در زبان انگلیسی مورد تایید است. همچنین میزان رضایت از پروتز فعلی افراد با گزینه های خیلی زیاد، زیاد، متوسط، کم و خیلی کم سنجیده شد. یافته ها:  5/88% افراد از حس خیالی، 77% از درد خیالی، 1/36% درد در اندام باقیمانده، 33% درد در اندام مقابل و 1/72% کمر درد داشتند. 67/19% افراد از پروتز فعلی خود رضایت خیلی زیاد را گزارش کردند. نتیجه گیری:  طبق مطالعه حاضر بیشترین شیوع درد در قطع اندام تحتانی به ترتیب مربوط به حس خیالی، درد اندام خیالی، کمر درد، درد اندام باقیمانده و درد در اندام سمت مقابل می شود و بیش از نیمی از افراد از پروتز فعلی خود رضایت متوسط با بالا داشتند.

Title: بررسي رابطه‌ي عزت نفس و انگیزش پیشرفت در ورزشكاران موفق و ناموفق استان آذربایجان‌غربی

http://jrrs.mui.ac.ir/index.php/jrrs/article/view/161

مقدمه:‌ عزت نفس عامل رشد رواني افراد بوده و اثرات برجسته اي را در جريان فكري، احساسات، ارزش‌ها و هدف‌هاي فرد ايفا مي‌‌كند و باعث انگيزش پيشرفت در فرد مي‌شود. هدف از این پژوهش بررسی رابطه عزت نفس و انگیزش پیشرفت در ورزشکاران موفق و ناموفق استان آذربایجان‌غربی است. مواد و روش‌ها:‌ آزمودنی‌های تحقیق 200 نفر ورزشکار مرد و زن بودند که به دو گروه تقسیم شدند :100 ورزشکار موفق که در سال 1387 در مسابقات رسمی داخلی و یا بین المللی موفق به کسب مدال شده بودند و 100 ورزشکار ناموفق در کسب مدال. ابزار اندازه‌گیری تحقیق پرسش نامه انگیزش پیشرفت ورزشی (SOQ) گیل و دیتر (1988) و فرم تجدید نظر شده پرسشنامه عزت نفس کوپر- اسمیت (1978) بود . برای تجزیه و تحلیل داده ها از ضریب همبستگی پیرسون و آزمون تي مستقل در سطح معنی‌داری 05/0  α< استفاده شد. يافته ها: نتایج نشان داد بین عزت نفس و انگیزش پیشرفت در هر دو گروه رابطه معنی دار مثبت وجود دارد. همچنین نتایج آزمون تي مستقل تفاوت معنی‌داری را بین عزت نفس و انگیزش پیشرفت ورزشکاران موفق و ناموفق نشان داد یعنی عزت نفس و انگیزش پیشرفت ورزشکاران موفق در مقایسه با ورزشکاران ناموفق به طور معنی داری بالاتر بود. نتيجه گيري: به نظر مي‌رسد باورهاي ورزشكاران در عملكردشان تأثير مي‌گذارد. بنابراين جهت دستيابي به آمادگي بدني و مهارت‌هاي ورزشي از طريق برنامه‌ریزی‌های صحیح در جلسات تمريني و فراهم کردن شرایط آموزشی به گونه‌ای که ورزشکاران در تجربیات حرکتی خود به موفقیت برسند، باعث می‌شود که ورزشکار خود را با کفایت احساس کند و این احساس کفایت از مهمترین متغیرهای انگیزشی است كه سبب افزایش انگیزش پیشرفت در ورزشکار می شود. بنابراین به مربیان توصیه مي‌شود با بازخورد و تقویت‌های به موقع و حمایت‌های ضروری و انتقادهای سازنده، موجبات آگاهی ورزشکاران از توانایی‌های خود را فراهم نمايند تا از این طریق سبب افزایش انگیزش پیشرفت آنها شوند.

Title: تاثیر غنی سازی ادراکی-حرکتی اولیه بر رشد بعدی حرکات درشت شیرخواران

http://jrrs.mui.ac.ir/index.php/jrrs/article/view/162

مقدمه:  پژوهش حاضر، با هدف تعیین تاثیر غنی سازی ادراکی-حرکتی اولیه  بر رشد بعدی حرکات درشت شیرخواران بود. روش بررسی:بدین منظور 15 شیرخوار 5 تا 8 ماهه سالم به صورت تصادفی از مرکز شیرخوارگاه آمنه شهر تهران انتخاب شدند و به صورت همگن از نظر سنی در گروه های تمرین(8 نفر، با میانگین سنی2±5/6 )و بی تمرین(7 نفر، با میانگین سنی2±5/6) قرار گرفتند. شیرخواران گروه آزمایش به مدت 36 جلسه (3 جلسه در هفته) و هر جلسه 60 دقیقه  در محیط غنی شده اداراکی-حرکتی(شامل  در دسترس بودن ابزار متنوع دستکاری و جابجایی) قرار گرفتند. پس از پایان 36 جلسه ،در مرحله اول اندازه‌گیری، مهارت های حرکتی درشت شیرخواران با استفاده از مقیاس رشد حرکتی پی بادی ارزیابی شد. سه ماه بعدی کلیه شیرخواران در شرایط یکسان و عادی نگهداری شدند. در مرحله دوم ،پس از گذشت این سه ماه،  مهارت های حرکتی درشت شیرخواران و مجددا با استفاده از مقیاس رشد حرکتی پی بادی ارزیابی شدند. داده های حاصل با استفاده از آزمون تحلیل واریانس عاملی مرکب (زمان)2×(گروه)2 مورد تجزیه و تحلیل قرار گرفت. یافته‌ها: نتایج نشان داد، در متغیر معادل سنی حرکات درشت ، اثر اصلی گروه( 50/15= 10و1F و 003/0P=)  و اثر اصلی مراحل اندازه‌گیری ( 95/56= 10و1F و 001/0P=) معنادار بود. تعامل میان گروه و مراحل اندازه‌گیری (95/1= 10و1F و 19/0P=) معنادار نبود. همچنین نتایج نشان داد، در متغیر نمره مقیاس بندی شده، اثر اصلی گروه (53/8= 10و1F و 015/0P= ) و اثر اصلی مراحل اندازه‌گیری( 60/151= 10و1F و 001/0P= ) معنادار بود. تعامل میان گروه و مراحل اندازه‌گیری( 66/0= 10و1F و 43/0P=) معنادار نبود. نتیجه گیری: به طور کلی، نتایج پژوهش حاضر نشان می دهد ، تجربه ادراکی-حرکتی اولیه  بر رشد حرکات درشت  بعدی شیرخواران تاثیر مثبتی دارد.

Title: تاثير تمرينات پيلاتس بر درد و سلامت عمومي بيماران زن داراي کمردرد مزمن

http://jrrs.mui.ac.ir/index.php/jrrs/article/view/163

مقدمه: شواهد زيادي وجود دارد که  ورزش درماني براي بهبود بيماران داراي کمردرد مزمن مؤثر است. يكي از روش هاي ورزش درماني كه در سال هاي اخير مورد توجه متخصصين ورزشي و توانبخشي قرار گرفته است و به طور وسيعي در حال فراگير شدن مي باشد، ورزش پيلاتس است.  هدف از اين مطالعه بررسي تاثير تمرينات پيلاتس بر ميزان درد و سلامت عمومي  بيماران زن داراي  کمردرد مزمن مي باشد. مواد و روشها: در اين مطالعه 28 بيمار زن مبتلا به کمردرد مزمن، در دو گروه تجربي(15 بيمار با ميانگين سنی 8.07±40.20) و کنترل (13 بيمار با ميانگين سنی 7.84±38.30) قرار داده شدند. گروه تجربي به مدت 6 هفته تمرينات تعديل شده پيلاتس، و گروه کنترل در مدت زمان مشابه، درمان­هاي رايج را دريافت کردند. شدت درد و ميزان سلامت عمومي تمامي آزمودني­ها به ترتيب توسط شاخص اندازه گيري ميزان درد[1] و پرسشنامه سلامت عمومي (GHQ28)[2] اندازه گيري شدند. براي تجزيه و تحليل داده ها از آزمون تحليل کوواريانس، با عامل کووريت (پيش­آزمون) در سطح =0.05α استفاده گرديد. يافته­ها: نتايج نشان داد که پس از 6 هفته درمان، ميانگين شاخص درد (024/0p=) گروه تجربي کمتر از گروه کنترل، بود. همچنين ميزان سلامت عمومي گروه تجربي بهبود بيشتر را نسبت به گروه کنترل نشان داد (04/0p=) . بحث و نتيجه­گيري: با توجه به يافته­هاي اين پژوهش، تمرينات پيلاتس، در بهبود درد و سلامت عمومي، مؤثرتر از روش­هاي درماني رايج براي بيماران مبتلا به کمردرد مزمن مي­باشد.  يافته هاي تحقيق حاضر،از توصيه تمرينات پيلاتس به بيماران مبتلا به کمردرد مزمن حمايت مي کند.

Title: ميزان پذيرش پروتز در آمپوته هاي اندام فوقاني و ارتباط آن با نوع پروتز و سطح قطع عضو

http://jrrs.mui.ac.ir/index.php/jrrs/article/view/164

قطع عضو اندام فوقاني باعث از دست رفتن بخش وسيعي از توانائي فرد درانجام فعاليت هاي مختلف مي شود که براي جبران آن از انواع مختلف پروتز استفاده مي شود. انواع مختلفي از پروتزهاي اندام فوقاني طراحي وساخته  شده است که با توجه به مکانيزم هاي حرکتي مي توان آنها را به پروتزهاي کازميک، مکانيکال و مايوالکتريک تقسيم کرد. متاسفانه ميزان پذيرش پروتز دربين آمپوته هاي اندام فوقاني بسيار پايين بوده كه اين امر به عوامل مختلفي بستگي دارد. در اين تحقيق سعي بر آن است که تاثير عواملي همچون سطح قطع عضو ، نوع پروتز ومدت زمان استفاده از آنرا برميزان پذيرش پروتز مورد برسي قرار دهيم . روش: تعداد 76 نفر از آمپوته هاي اندام فوقاني در شهرستانهاي اصفهان , تهران, شيراز موردتحقيق قرارگرفتند براي ارزيابي ميزان پذيرش پروتز از يک پرسشنامه استاندارد استفاده گرديد. نتايج: نتايج اين تحقيق نشان داد که بين ميزان پذيرش پروتز و سطح قطع عضو ارتباط معني داري وجود ندارد؛ با اين حال ميزان پذيرش پروتز دربين آمپوته هاي سطوح مچ و بالاي آرنج نسبت  به سطوح ديگر کمتر است. نوع پروتز  بر ميزان کارائي آن تاثير قابل ملاحظه اي ندارد؛ چرا که افراد اکثراً از پروتز درحد يک تکيه گاه استفاده مي کنند. بحث ونتيجه گيري: با توجه به اينکه ميزان پذيرش پروتز درميان آمپوته هاي اندام فوقاني پائين مي باشد، بايستي درجهت بالا بردن زبيائي پروتز و افزايش کارائي آن تلاش کرد.

Title: بررسی ارتباط مهارت آگاهی واجی وعملکرد ریاضی در دانش آموزان پايه اول دبستان

http://jrrs.mui.ac.ir/index.php/jrrs/article/view/153

مقدمه:   یادگیری عددی یکی از اجزای اساسی آموزش است ونقص در ادراک ریاضی نه تنها عملکرد در مدرسه بلکه عملکرد زندگی روزمره را آسیب می رساند. مطالعات تجربی کمی در زمینه ی فاکتورهای شناختی موثر بر مهارت ریاضی به ویژه در میان کودکان دبستانی انجام شده است.لذا لازم است فاکتورهای شناختی زیر بنایی در رشد توانایی ریاضی در جنبه های مختلف مورد بررسی قرار بگیرد وازآنجاییکه  در حل مسائل این حوزه ی تحصیلی از پردازش صداهای گفتاری استفاده می شود، آگاهی واجشناختی می تواند از جمله این  فاکتورها باشد.  مواد وروشها: این مطالعه  توصیفی تحلیلی ومقطعی بود و70دانش آموز پایه اول که دارای هوش طبیعی بوده وسابقه مردودی در پایه اول ومشکلات شنوایی وزبانی نداشتتند به صورت تصادفی انتخاب شدندوتحت آزمون آگاهی واجی وآزمون ریاضات ایران کی مت قرار گرفتند. پس از اجرای آزمون برطبق دفترچه ی راهنما امتیازهای آن در برگه های مربوطه ثبت شد و اطلاعات حاصل با استفاده از نرم افزار SPSSمورد تجزیه وتحلیل قرار گرفت.     یافته ها: مهارت کل آگاهی واجی و کل عملکرد ریاضی با یکدیگر ارتباط دارند(ضریب همبستگی 947/0)وهمچنین بین نمره ی هر یک ا ز خرده آزمونهای مهارت آگاهی واجی و نمره ی هر یک از خرده آزمونهای عملکرد ریاضی در نمونه مورد بررسی ارتباط معناداری وجود داردو بیشترین ارتباط مربوط به  حذف واج میانی وشمارش با ضریب همبستگی    74/0  و کمترین ارتباط مربوط به  تجزیه واجی و هندسه  با ضریب همبستگی 42/0  می باشد.  بحث:   نتایج پژوهش حاضر با سایرپژوهش های انجام شده همخوانی دارد ضریب همبستگی میان دو متغییر در پژوهش های انجام شده متفاوت است که می توان علت را به تفاوت در تعداد متغییر های مورد پژوهش ،تقسیم بندی متفاوت از مهارت آگاهی واجی و عملکرد ریاضی ،آزمون های متفاوت استفاده شده در پژوهش ها نسبت داد.
